# Kidney Transplantation Improves Health-Related Quality of Life in Older Recipients

**DOI:** 10.3389/ti.2024.12071

**Published:** 2024-04-15

**Authors:** Silke E. de Boer, Tim. J. Knobbe, Daan Kremer, Barbara C. van Munster, Gertrude J. Nieuwenhuijs-Moeke, Robert A. Pol, Stephan J. L. Bakker, Stefan P. Berger, Jan Stephan F. Sanders

**Affiliations:** ^1^ Department of Internal Medicine, Division of Nephrology, University Medical Center Groningen and University of Groningen, Groningen, Netherlands; ^2^ Department of Internal Medicine, Division of Geriatrics, University Medical Center Groningen, University of Groningen, Groningen, Netherlands; ^3^ Department of Anesthesiology, University Medical Center Groningen and University of Groningen, Groningen, Netherlands; ^4^ Department of Surgery, University Medical Center Groningen and University of Groningen, Groningen, Netherlands

**Keywords:** elderly, health-related quality of life, kidney transplantation, immunosuppression, patient reported outcome measures, side-effects

## Abstract

Kidney transplantation is the best treatment for kidney failure in older patients. However, little is known regarding changes in health-related quality of life (HRQoL) from before to after transplantation and determinants of HRQoL in older kidney transplant recipients (KTR). We studied both, using data of older (≥65 years) patients waitlisted for kidney transplantation and older KTR 1 year after transplantation from the TransplantLines Biobank and Cohort Study. HRQoL was assessed using the SF-36 questionnaire. We included 145 older waitlisted patients (68% male, age 70 ± 4 years) and 115 older KTR at 1 year after transplantation (73% male, age 70 ± 4 years). Both mental (48.5 ± 8.4 versus 51.2 ± 7.7, *p* = 0.009) and physical (47.4 ± 8.5 versus 52.1 ± 7.2, *p* < 0.001) HRQoL were higher among included KTR, compared to the waitlisted patients. In paired analyses among 46 patients with HRQoL-data both before and after transplantation, there was a trend towards increased mental HRQoL (49.1 ± 8.4 to 51.6 ± 7.5, *p* = 0.054), and significantly increased physical HRQoL (48.1 ± 8.0 to 52.4 ± 6.7, *p* = 0.001) after transplantation. Among all assessed factors, the number of patient-reported immunosuppressive drug-related side effects was most strongly negatively associated with both mental and physical HRQoL. In conclusion, HRQoL is significantly higher among older KTR after kidney transplantation compared to older waitlisted patients.

## Graphical Abstract

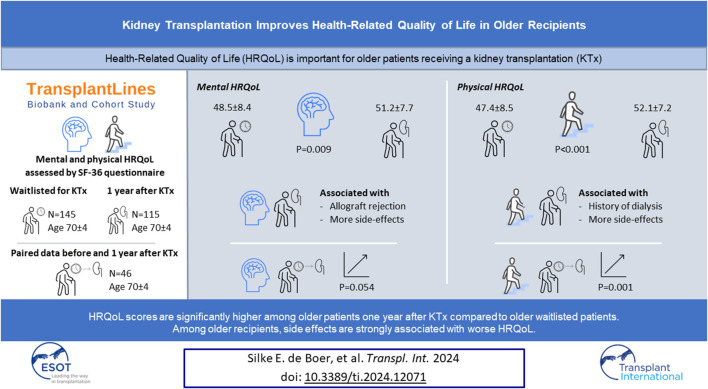

## Introduction

Kidney transplantation is the preferred treatment for patients with end-stage kidney disease. It improves quality of life and offers survival benefit in comparison with other forms of kidney replacement therapy [1]. Older patients are an important and growing part of the kidney transplant population. In 2019, 30% of the newly transplanted kidney transplant recipients (KTR) in the Netherlands were above 65 years of age, compared to ∼15% in 2005 [2]. Most studies show at least some survival benefit for older KTR in comparison with patients that remain on the waiting list [3–6], while some other studies show no survival benefit [7–10]. Importantly, several studies indicate that the mortality risk for older KTR is up to 3 times higher in the first 3–12 months after kidney transplantation, in comparison with waitlisted patients [4, 7, 11].

Kidney transplantation, however, is also associated with improvement in health-related quality of life (HRQoL) [1]. Because the survival gain is limited among older patients, any improvement in HRQoL may be an important reason to consider a kidney transplantation in this population. Unfortunately, data on HRQoL of older KTR are scarce and most previous studies only report data from small cohorts (<55 patients) [12, 13]. In addition, a comparison of HRQoL before and after transplantation was studied in only one population of older KTR thus far [14, 15]. Consequently, factors associated with HRQoL in older KTR remain largely unknown, even though these may help to identify patients that could benefit most from transplantation.

The aim of this study was to fill the important knowledge gaps that now exist regarding HRQoL before and after kidney transplantation of the growing and distinct group of older patients. Such information is crucial for providing proper counselling to older patients for renal replacement therapy. To do so, we compared HRQoL of older (≥65 years) patients waitlisted for kidney transplantation with HRQoL of older KTR 1 year after transplantation. In addition, we aimed to identify potential determinants of HRQoL after kidney transplantation.

## Patients and Methods

### Study Design

We used data from the ongoing, prospective, TransplantLines Biobank and Cohort study (ClinicalTrials.gov identifier: NCT03272841) [16]. From June 2015, all (potential) solid organ transplant patients and donors (aged ≥18 years) of the University Medical Centre Groningen (UMCG, Netherlands) were invited to participate. All participants gave written informed consent on enrolment. The study protocol was approved by the local Institutional Review Board (METc 2014/077), adheres to the UMCG Biobank Regulation, and is in accordance with the WMA Declarations of Helsinki. The clinical and research activities being reported are consistent with the Principles of the Declaration of Istanbul as outlined in the “Declaration of Istanbul on Organ Trafficking and Transplant Tourism.”

### Study Population

**FIGURE 1:**
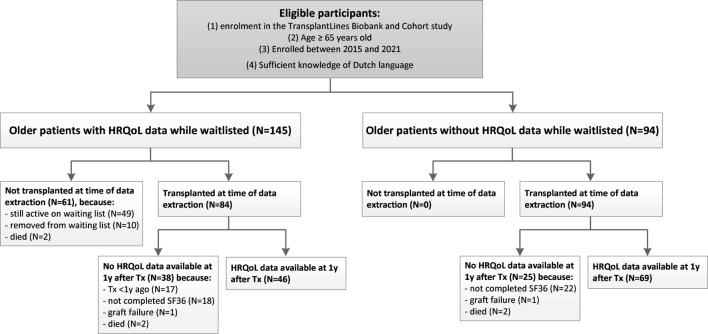
Patient flow diagram. Abbreviations: HRQoL, health-related quality of life; KTR, kidney transplant recipients; Tx, transplantation.

The participation rate of the TransplantLines Biobank and Cohort study was 81%. We included HRQoL data of 145 older (≥65 years at the time of evaluation) patients waitlisted for kidney transplantation. HRQoL was assessed after transplant evaluation and approval, but before transplantation. In addition, HRQoL data of 115 older (≥65 years at the time of transplantation) KTR 1 year after transplantation were included. Of note, within these groups, 46 patients had available HRQoL both before and after transplantation, and were therefore included in both groups. A flow diagram is presented in Figure 1. As can be depicted from Figure 1, there is a group of older patients without HRQoL data while waitlisted. The main reason therefore is that they were included in the Transplantlines Biobank and Cohort study shortly after transplantation. Furthermore, it is important to denote that all KTR were in close medical follow-up after transplantation, also the ones that did not complete the SF-36 1 year after transplantation.

### Assessment of HRQoL

HRQoL was assessed using a Dutch translation of the 36-item Short Form-36 (SF-36) Health Survey [17]. Higher scores reflect better HRQoL [17]. Using reference values of the Dutch population [17], aged 65–75 years, a standardized mental component score (MCS) and physical component score (PCS) were calculated [18]. By definition, this implies that if a participant has the same HRQoL score as the presented reference value, their score is 50. Generally, scores between 45–55 are considered to be average, and scores <40 are indicative of significantly impaired HRQoL [19].

### Assessment of Covariables

Demographic and clinical data—including participation in the Eurotransplant Senior Program—were retrieved from the medical files. The Eurotransplant Senior Program allocates kidneys within a geographic area from donors aged ≥65 years to recipients ≥65 years regardless of HLA match, thereby minimizing cold ischemia time [20]. Medical comorbidities at time of evaluation or transplantation were indexed using the Charlson Comorbidity Index [21]. Because all older patients received 2 points for the presence of their kidney disease, a Charlson Comorbidity Index score of 2 means that there are no other indexed comorbidities. Delayed graft function was defined as the need for dialysis in the first week after transplantation, and a rejection episode as a treated biopsy-proven acute rejection. Participants were defined as having post-transplant diabetes mellitus when they did not meet the diabetes mellitus criteria set by American Diabetes Association before transplantation, but did meet these criteria at 1 year after transplantation [22, 23]. Malnutrition was defined according to the criteria set by the Global Leadership Initiative on Malnutrition, considering that all KTR meet the etiologic criteria for disease burden/inflammation [24]. Data regarding partner status, children, education and financial situation were obtained from questionnaires. Number of immunosuppressive-drug related side effects was assessed using the Modified Transplant Symptom Occurrence and Symptom Distress Scale-59R at 1 year after transplantation [25]. Side-effects were considered as present if the symptom occurred regularly, almost always or always. Covariables that were selected had previously been found to be associated with HRQoL [26–32].

### Statistical Analyses

Normally distributed data are presented as mean ± standard deviation (SD), non-normally distributed data as median [interquartile range [IQR]] and categorical data as numbers (valid percentages). Statistical difference between two groups were assessed using one-sample T-tests, independent sample T-tests, Mann-Whitney U tests, and Fisher’s exact test or Pearson Chi-Square tests for categorical variables. Additionally, differences in HRQoL scores of older patients waitlisted for kidney transplantation and older KTR 1 year after transplantation were assessed using Cohen’s D effect size. This effect size was calculated by dividing the difference between the pre- and posttransplant score by the (pooled—for independence groups) SD of this difference. Effect sizes of 0.2, 0.5 and 0.8 are considered as small, medium and large, respectively [33].

Among KTR with data both before and 1 year after transplantation, differences in HRQoL scores were assessed using paired sample T-tests. Furthermore, within this subgroup, we compared the characteristics of older KTR with a MCS or PCS below the median score before transplantation with those of older KTR with a MCS or PCS above the median score before transplantation. Finally, we assessed which factors are associated with HRQoL of older KTR at 1 year after transplantation using univariable and multivariable linear regression analyses adjusted for age, sex and pre-emptive transplantation with the MCS and PCS as dependent variables. The variance inflation factor was measured for each variable in the multivariable regression analysis and was <1.7 in all analyses [34].

Data were analyzed using SPSS software version 23.0 and R version 4.1.1. In all analyses, a two-sided *p*-value <0.05 was considered as statistically significant.

## Results

### Baseline Characteristics

In total, 145 patients waitlisted for kidney transplantation (age 70 ± 4 year; 68% males) were included in the study, among whom 65 patients (45%) were on dialysis at the time of HRQoL assessment. The median Charlson Comorbidity Index in this population was 3 [IQR 2–4].

In addition, 115 older KTR (age at time of transplantation 70 ± 4 year; 72% males) with available data on HRQoL 1 year after transplantation were included, among whom 46 (40%) were pre-emptively transplanted. Notably, half of the older KTR had a Charlson Comorbidity Index of 2 at the time of the transplantation, indicating no other indexed comorbidities besides end-stage kidney disease. More extensive baseline characteristics of both populations are shown in Table 1. Clinical characteristics of older waitlisted patients and older KTR were comparable (Table 1).

**TABLE 1:** Characteristics of older patients waitlisted for kidney transplantation and of older KTR one year after transplantation.

Patient demographics	Older patients waitlisted for KTx (*N* = 145)	Older KTR (*N* = 115)
Male sex, *n* (%)	98 (68)	83 (73)
Age at time of HRQoL assessment/Tx	70 ± 4	70 ± 4
White patients, *n* (%)	140 (97)	110 (96)
BMI, kg/m^2^	27 ± 4	27 ± 4
Partner at time of HRQoL assessment/Tx, *n* (%)	122 (84)	95 (83)
Children, *n* (%)	130 (90)	104 (90)
Education level, *n* (%)		
Low	70 (49)	43 (40)
Intermediate	32 (22)	30 (28)
High	42 (29)	35 (32)
Financial situation, *n* (%)		
(Some) shortage of money	5 (4)	3 (3)
Just right	32 (22)	27 (28)
(Some) money left	84 (59)	66 (69)
Does not want to tell	22 (15)	4 (4)
Medical history		
Dialysis before HRQoL assessment/Tx	65 (45)	69 (60)
Hemodialysis	55 (38)	46 (40)
Time on hemodialysis, weeks	86 ± 76	109 ± 63
Peritoneal dialysis	10 (7)	23 (20)
Time on peritoneal dialysis, weeks	76 ± 62	96 ± 50
None	80 (55)	46 (40)
Diabetes mellitus at time HRQoL assessment/Tx	45 (31)	17 (15)
Charlson Comorbidity Index score	3 [2–4]	3 [2-3]
Primary kidney disease, *n* (%)		
Glomerulonephritis	20 (14)	19 (17)
Interstitial nephritis	10 (7)	8 (7)
Cystic kidney disease	23 (16)	17 (15)
Other congenital/hereditary disease	4 (3)	1 (1)
Renal vascular disease, excluding vasculitis	35 (24)	26 (23)
Diabetic nephropathy	19 (13)	7 (6)
Other multisystem diseases	9 (6)	8 (7)
Other	4 (3)	7 (6)
Unknown	21 (15)	22 (19)
Transplant-specific characteristics		
First kidney Tx, *n* (%)	140 (97)	106 (93)
Other organ Tx before kidney Tx, *n* (%)	1 (1)	2 (2)
Donor type, *n* (%)		
Donation after cardiac death	n/a	20 (17)
Donation after brain death	n/a	41 (36)
Living donor	n/a	54 (47)
Eurotransplant Senior Program donor, *n* (%)	n/a	42 (37)
ABO-incompatible transplantation, *n* (%)	n/a	7 (6)
Donor sex, *n* males (%)	n/a	64 (56)
Donor age, years	n/a	66 [62–70]
Induction therapy, *n* (%)		
Basiliximab	n/a	108 (94)
Antithymocyte globulin	n/a	2 (2)
Alemtuzumab	n/a	2 (2)
Rituximab	n/a	7 (6)
None	n/a	3 (3)
Immunosuppressive starting regimen, *n* (%)		
TAC/MMF/prednisolone	n/a	108 (94)
Cyclosporine/MMF/prednisolone	n/a	2 (2)
TAC/EVR/prednisolone	n/a	4 (4)
Iscalimab (CFZ-533)/MMF/prednisolone	n/a	1 (1)

Data regarding educational level and financial situation were missing in, 1 and 2 older patient(s) waitlisted for KTx and in 7 and 15 older KTR, respectively. Other variables were complete.

Normal distributed variables are presented as mean ± SD, not-normally distributed variables as median [IQR] and categorical data as number (valid %).

Abbreviations: EVR, everolimus; HRQoL, Health related quality of life; KTR, kidney transplant recipients; (K)Tx, (kidney)transplantation; MMF, mycophenolic acid; TAC, tacrolimus.

### Course of the First Year After Transplantation

Among the 115 older KTR with available data on HRQoL 1 year after transplantation, mean eGFR 1 year after transplantation was 48 ± 16 mL/min/1.73 m^2^. Fifteen older KTR (13%) had an eGFR ≤30 mL/min/1.73 m^2^. Nine older KTR (8%) suffered from rejection in the first year. Forty-two (37%) older KTR were malnourished and 20 (17%) developed post-transplant diabetes mellitus (PTDM) 1 year after transplantation.

During the first year, 49% of patients were hospitalized at least once in addition to the admission for transplantation. Main reasons for the total of 100 hospitalizations were infections (*N* = 47), elective surgeries (*N* = 15) and hemorrhage (*N* = 8). In addition, we observed 9 hospitalizations for (analysis of) kidney function decline, including rejection.

A detailed overview of clinical outcomes and events among older KTR in the first year after transplantation is presented in Table 2.

**TABLE 2:** Clinical outcomes one year after transplantation for 115 older KTR with available data on HRQoL one year after transplantation.

Clinical course	Older KTR (*N* = 115)
eGFR at one year after Tx, mL/min/1.73 m^2^	48 ± 16
eGFR <30 mL/min/1.73 m^2^ at one year after Tx, *n* (%)	15 (13)
Hemoglobin, g/dL	13.2 ± 1.6
Delayed graft function, *n* (%)	26 (23)
Rejection in the first year after Tx, *n* (%)	9 (8)
Methylprednisolone	7 (6)
Antithymocyte globulin	4 (3)
Alemtuzumab	1 (1)
Malnourished at one year after Tx, *n* (%)	42 (43)
Development of PTDM during the first year after Tx, *n* (%)	20 (17)
Malignancy during the first year after Tx, *n* (%)	7 (6)
BCC and/or SCC (single or multiple)	4
Other (colorectal, prostate, breast, bladder)	3^a^
Cardiovascular event during the first year after Tx, *n* (%)	3 (3)
Number of hospitalizations in the first year after Tx per older KTR, *n* (%)	
No hospitalization	59 (51)
1 hospitalization	30 (26)
2 hospitalizations	19 (17)
≥ 3 hospitalizations	7 (6)
Number of infections in the first year after Tx per older KTR^b^, *n* (%)	
No infection	47 (41)
1 infection	30 (26)
2 infections	18 (16)
≥ 3 infections	20 (17)
CMV-primo infection in the first year after Tx, *n* (%)	7 (6)
BK viraemia in the first year after Tx, *n* (%)	25 (22)

Data regarding malnourishment were missing in 17 older KTR. Other variables were complete.

Normal distributed variables are presented as mean ± SD, not-normally distributed variables as median [IQR] and categorical data as number (valid %).

Abbreviations: BCC, Basal cell carcinoma; eGFR, estimated glomerular filtration rate; KTR, kidney transplant recipient; SCC, Squamous cell carcinoma.

^a^ One patient had a SCC and other cancer.

^b^ Excluding CMV and BK viraemia.

The baseline characteristics of the 40 patients without HRQoL data at 1 year after transplantation were comparable to the 115 patients included in analyses, with the exception for the prevalence of diabetes, which was higher among patients without HRQoL data at 1 year after transplantation. Notably, there was no significant difference in either mental or physical HRQoL prior to transplantation between both groups (Supplementary Tables S1, S2).

### Reported Side Effects of Immunosuppressive Drugs One Year After Transplantation

Self-reported immunosuppressive-drug related side effects at 1 year after transplantation were common; the median number of side effects was 4 [IQR: 2–9] per KTR. Notably, 10 (9%) older KTR reported no side-effects. The most reported side-effects were erectile dysfunction (46% of males), bruises (36%), tremor (30%), dry skin (26%), reduced interest in sex (25%), increased urge to urinate (22%) and lack of energy (23%).

### HRQoL of 145 Older Patients Waitlisted for Kidney Transplantation

**FIGURE 2:**
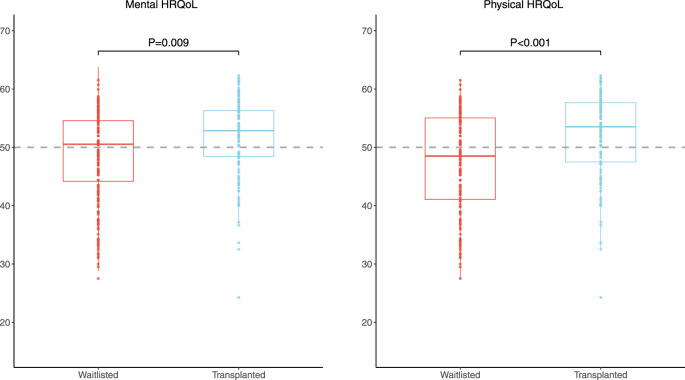
Boxplot of standardized MCS and PCS of 145 older patients waitlisted for kidney transplantation and 115 older KTR 1 year after transplantation. The dotted grey line represents the age-matched general population, which by definition has standardized PCS and MCS scores of 50.0.

Mean mental (standardized MCS 48.5 ± 8.4) and physical HRQoL (standardized PCS 47.4 ± 8.5) scores of 145 older waitlisted patients were lower than the age-matched general population (*p* = 0.037 and *p* < 0.001, respectively), of which the mean score is by definition 50.0 ± 10.0 (Figure 2).

Among the HRQoL subdomains, the score on the mental health subdomain of the waitlisted patients was higher in comparison with the age-matched general population (78.9 ± 13.5 versus 75.9 ± 17.3; *p* = 0.009). The score on the physical functioning subdomain did not differ between waitlisted patients and the age-matched general population (*p* = 0.33). Scores on all other 6 subdomains were significantly lower among waitlisted patients compared to the scores of the age-matched general population (*p* < 0.05 for all, Supplementary Table S3).

Mean mental and physical HRQoL scores of the 12 waitlisted patients that did not receive a transplantation (because they were delisted or died on the waiting list) did not differ from those of the 84 patients that got a transplantation (standardized MCS 52.3 (±7.7) versus 48.2 (±8.3, P for difference 0.107), standardized PCS 44.3 (±11.1) versus 46.6 (±8.4, P for difference 0.502).

### HRQoL of 115 Older Patients After Kidney Transplantation

Mean mental HRQoL of 115 older KTR at 1 year after transplantation was comparable to the age-matched general population (standardized MCS: 51.2 ± 7 versus 50.0 ± 10.0 *p* = 0.10), while mean physical HRQoL was significantly higher (standardized PCS: 52.1 ± 7.2 versus 50.0 ± 10.0, *p* = 0.003) (Figure 2). Older KTR at 1 year after transplantation had better scores on the HRQoL subdomains “mental health” (*p* < 0.001), “vitality” (*p* = 0.035), “bodily pain” (*p* < 0.001) and “physical functioning” (*p* < 0.001) compared to the age-matched general population. Other subdomain scores were comparable (*p* > 0.05, Supplementary Table S3).

### Comparison of HRQoL of 145 Older Patients Waitlisted for Kidney Transplantation and 115 Older Patients After Kidney Transplantation

**FIGURE 3:**
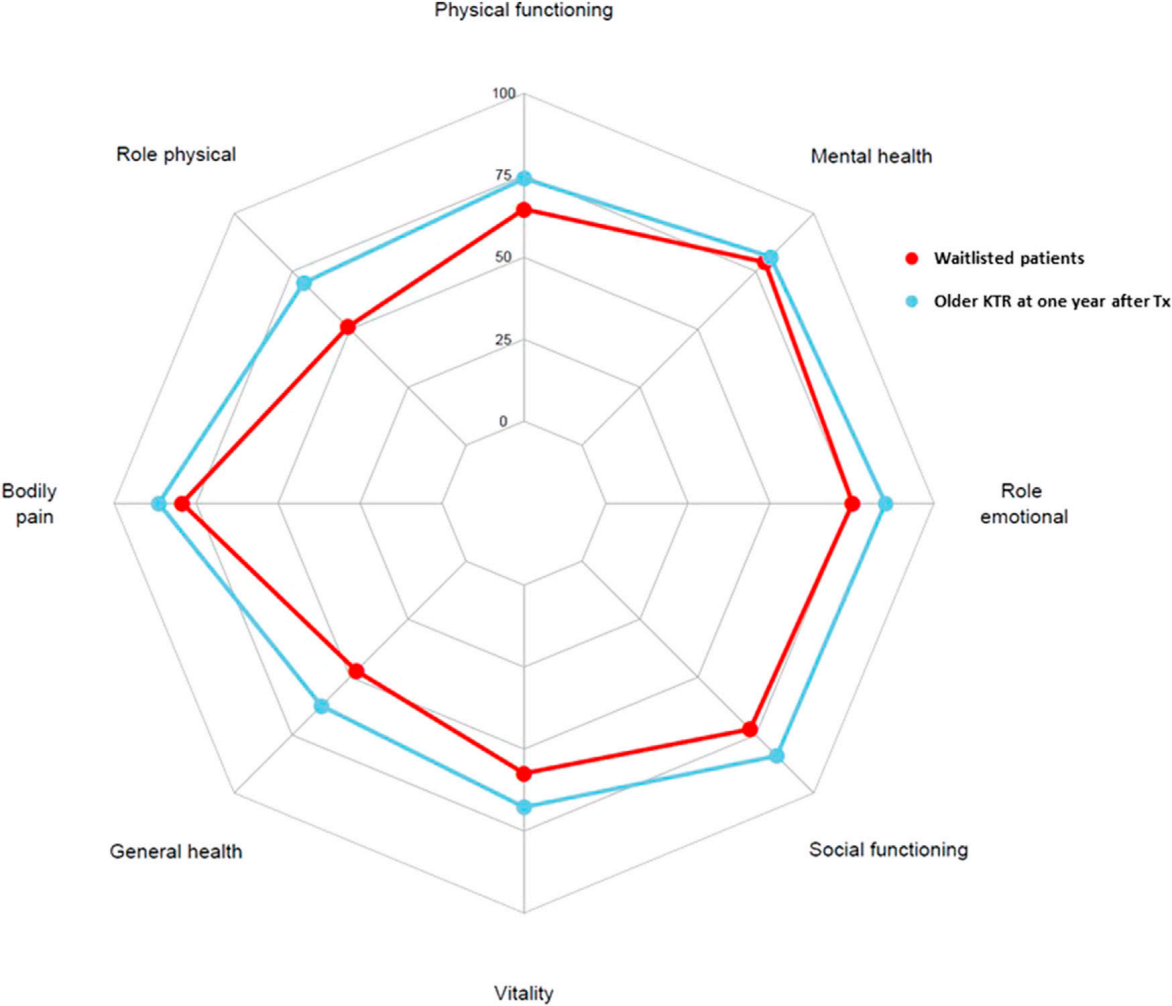
Comparison of HRQoL subdomain scores of 145 older patients waitlisted for kidney transplantation (blue) with HRQoL subdomain scores of 115 older KTR at 1 year after transplantation (red). All HRQoL subdomain scores were higher among older KTR 1 year after transplantation compared to older patients waitlisted for transplantation (*p* < 0.02 for all), with the exception for the mental health subdomain, which was not statistically different.

Both mental and physical HRQoL scores were significantly higher among 115 older KTR at 1 year after transplantation compared to 145 older waitlisted patients (standardized MCS: 51.2 ± 7.7 versus 48.5 ± 8.4, *p* = 0.009, Cohen’s D 0.32 and standardized PCS: 52.1 ± 7.2 versus 47.4 ± 8.5, *p* < 0.001, Cohen’s D 0.59), as presented in Figure 2. In addition, all HRQoL subdomain scores, except the subdomain “mental health,” were significantly higher among older KTR at 1 year after transplantation compared with older waitlisted patients (*p* < 0.02 for all) (Figure 3; Supplementary Table S4).

Mental and physical HRQoL among patients waitlisted for transplantation was not different for patients <70 or ≥70 years old. Similarly, 1 year post-transplantation, there was no difference in HRQoL among between these two age categories (data not shown).

### Comparison of HRQoL Before and After Transplantation Among 46 Patients With Repeated HRQoL Measurements

In paired analyses among 46 older KTR with HRQoL data both from the waitlist period and at 1 year after kidney transplantation, mental HRQoL scores after transplantation were numerically higher, but not statistically different (standardized MCS: 49.1 ± 8.4 versus 51.6 ± 7.5, *p* = 0.05, Cohen’s D 0.29), as presented in Supplementary Table S5. Physical HRQoL scores were significantly higher after transplantation compared to before transplantation (standardized PCS: 48.1 ± 8.0 versus 52.4 ± 6.7, *p* = 0.001, Cohen’s D 0.52). Results of subdomain scores were generally comparable to the unpaired analyses.

**FIGURE 4:**
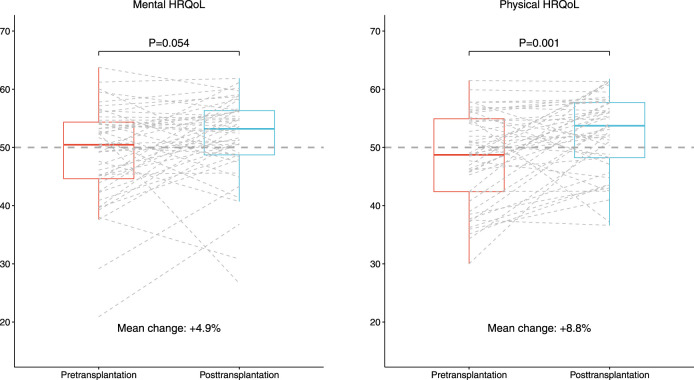
Boxplot of standardized MCS and PCS of 46 older KTR with HRQoL data both before and after transplantation. The dotted grey line represents the age-matched general population, which by definition has standardized PCS and MCS scores of 50.0.

Notably, the largest HRQoL improvements were observed in patients with a HRQoL lower than median HRQoL score before transplantation (difference in standardized MCS for patients below and above median: +7.6 ± 7.0 and −2.6 ± 6.7, *p* < 0.001, respectively). This difference was more pronounced regarding the physical HRQoL score (difference in standardized PCS for patients below and above median: +9.5 ± 6.8 and −1.0 ± 6.0, *p* < 0.001, respectively), as presented in Figure 4; Supplementary Table S6. Characteristics of KTR with HRQoL scores before transplantation below or above the median were not statistically different.

### Factors Associated With HRQoL of Older KTR at One Year After Transplantation

In linear regression analyses, after adjustment for age, sex and pre-emptive transplantation, number of patient-reported immunosuppressive-drug related side effects was strongly associated with a lower mental HRQoL at 1 year after transplantation (St. β −0.36, 95% CI −0.55 to–0.18). Other associated factors with mental HRQoL were rejection in the first year (St. β −0.33, 95% CI −0.51 to −0.15) and hospitalization in the first year after transplantation [Table 3; Supplementary Table S7 (for crude values)].

**TABLE 3:** Factors associated with mental and physical HRQoL at one year after transplantation among older KTR.

	Linear regression analyses with standardized MCS as dependent variable			Linear regression analyses with standardized PCS as dependent variable		
	Model 1			Model 1		
**Variables**	**St. β**	**95% CI**	**P**	**St. β**	**95% CI**	**P**
**Patient demographics**						
Female sex	−0.18	−0.36 to 0.01	0.07	−0.02	−0.20 to 0.17	0.85
Age	0.05	−0.14 to 0.24	0.59	-0.03	−0.22 to 0.15	0.73
BMI	0.12	−0.07 to 0.30	0.20	0.04	−0.14 to 0.23	0.66
Partner	0.13	−0.06 to 0.32	0.18	−0.06	−0.26 to 0.13	0.54
Children	−0.03	−0.21 to 0.16	0.79	0.01	−0.17 to 0.19	0.92
Education level						
Low	−0.18	−0.42 to 0.06	0.15	0.02	−0.23 to 0.27	0.88
Intermediate		reference			reference	
High	−0.12	−0.35 to 0.11	0.31	−0.01	−0.25 to 0.23	0.94
Financial situation						
(Some) shortage of money	0.02	−0.21 to 0.24	0.88	−0.03	−0.26 to 0.18	0.75
Just right		reference			reference	
(Some) money left	−0.09	−0.31 to 0.12	0.40	-0.24	−**0.46 to** −**0.03**	**0.027**
**Medical history**						
Pre-emptively transplanted	0.01	−0.18 to 0.19	0.92	0.25	**0.06 to 0.43**	**0.008**
Diabetes mellitus pre-KTx	0.06	−0.12 to 0.25	0.50	0.08	−0.11 to 0.26	0.40
CCI pre-KTx	−0.12	−0.31 to 0.06	0.20	-0.06	−0.25 to 0.12	0.50
CCI 3 or higher pre-Tx	−0.18	−0.37 to 0.00	0.06	−0.11	−0.30 to 0.07	0.23
**Kidney transplant characteristics**						
Living donor	0.01	−0.22 to 0.24	0.94	−0.18	−0.40 to 0.05	0.122
**Clinical course of the first year after Tx**						
eGFR	0.15	−0.03 to 0.34	0.10	−0.06	−0.24 to 0.13	0.57
eGFR <30 ml/min/1.73m^2^	−0.17	−0.35 to 0.01	0.07	−0.07	−0.25 to 0.11	0.45
Hemoglobin	0.14	−0.05 to 0.33	0.15	0.17	−0.02 to 0.35	0.083
Delayed graft function	−0.17	−0.38 to 0.03	0.09	−0.01	−0.21 to 0.20	0.95
Rejection in the first year after Tx	−0.33	−**0.51 to** −**0.15**	**<0.001**	−0.02	−0.20 to 0.17	0.87
Malnourished at one year after Tx	0.08	−0.12 to 0.27	0.48	−0.19	−0.39 to 0.01	0.07
Development of PTDM during the first year after Tx	−0.13	−0.31 to 0.06	0.18	−0.22	−**0.40 to** −**0.04**	**0.016**
Number of hospitalizations in the first year after Tx per older KTR	−0.21	−**0.40 to** −**0.02**	**0.030**	−0.12	−0.31 to 0.06	0.19
Any hospitalization in the first year after Tx	−0.24	−**0.42 to** −**0.05**	**0.014**	−0.14	−0.33 to 0.05	0.14
Number of infections in the first year after Tx per older KTR	−0.13	−0.32 to 0.05	0.15	−0.15	−0.33 to 0.03	0.09
Any infection in the first year after Tx	−0.09	−0.27 to 0.10	0.34	−0.12	−0.30 to 0.06	0.19
Number of side effects in the first year after Tx per older KTR	−0.36	−**0.55 to** −**0.18**	**<0.001**	-0.55	−**0.72 to** −**0.38**	**<0.001**

Model 1: All adjusted for age, sex, and pre-emptive tranplantation, except the variable age, sex and pre-emptive transplantation. Data regarding educational level was missing in 7 older KTR; 19 older KTR had missing data regarding financial situation or did not want to tell their financial situation. Abbreviations: MCS, mental component score of the SF-36 health-related quality of life questionnaire; PCS, physical component score of the SF-36 health-related quality of life questionnaire; CCI, Charlson Comorbidity Index; eGFR, estimated glomerular filtration rate; PTDM, post-transplant diabetes mellitus; Tx, transplantation.

Bold values represent statistically significant associations (*p* < 0.05).

Number of patient-reported immunosuppressive-drug related side effects was also strongly associated with lower physical HRQoL at 1 year after transplantation, after adjustment for age, sex and pre-emptive transplantation (St. β −0.55, 95% CI −0.72 to −0.38). Pre-emptive transplantation (St. β 0.25, 95% CI 0.06–0.43) was also positively associated. The mean physical HRQoL score of pre-emptive patients was significantly higher compared to patients with a dialysis vintage (standardized PCS: 54.2 ± 7.2 versus 50.6 ± 7.0, *p* = 0.011. Higher monthly income (St. β −0.24, 95% CI −0.46 to −0.03) and post-transplant diabetes mellitus (St. β −0.22. 95% CI −0.40 to–0.04) were negatively associated with physical HRQoL at 1 year after transplantation [Table 3; Supplementary Table S7 (for crude values)].

## Discussion

Our results show the beneficial effect of kidney transplantation on the health-related quality of life of older recipients. HRQoL at 1 year after transplantation of older KTR was higher compared to HRQoL of older patients waitlisted for kidney transplantation in both unpaired and paired analyses, and HRQoL of older KTR was equal or even better in comparison with the age-matched general population. Moreover, participants with the lowest HRQoL scores before transplantation showed the largest improvement of these scores after transplantation. Among all assessed factors, the number of patient-reported immunosuppressive-drug related side effects was the most important factor associated with both a lower mental and physical HRQoL, followed by an episode of allograft rejection for mental HRQoL and a history of dialysis for physical HRQoL.

A within-participant comparison of HRQoL before and after kidney transplantation among older patients has been previously performed in only one study population [14, 15]. Also in this population, a moderate to large HRQoL improvement at 1 year after transplantation compared to before transplantation was observed. This HRQoL improvement was already present at 2 months after transplantation [14], and persisted up to 3 years afterwards [14, 15]. Our findings are also in line with a previous study comparing HRQoL of older KTR with (non-waitlisted) hemodialysis patients [35]. Notably, we also confirmed that (general physical) HRQoL of older KTR is comparable to the age-matched population as previously reported [12, 36]. This might sound surprising, given the fact that a substantial part of the KTR had to deal with hospitalizations, infections and malnourishment in the first year after transplantation. It therefore is important to realize that HRQoL is a very subjective parameter.

Of note, as mentioned in the methods section, we used reference values from the Dutch population aged 65–75 years [17] to calculate the standardized MCS and PCS. This was chosen as it represents the most comparable population for our sample consisting of older Dutch patients. Since most other studies use reference values from the general U.S. population [18], the results of the MCS and PCS cannot readily be compared with those found in other studies.

Older KTR reported to experience a median of four side effects of immunosuppressive therapy, and the number of side effects was strongly associated with a lower HRQoL. This is in accordance with the findings of the BENEFIT (EXT) trial. This randomized-controlled trial showed that KTR treated with belatacept, which has generally less side-effects, had better HRQoL compared to calcineurin inhibitors-treated controls at several timepoints after transplantation [31]. We therefore hypothesize that alterations in the immunosuppressive regimen of older KTR may help to further improve their HRQoL, and this will be assessed in the ongoing OPTIMIZE-trial [37].

A history of treated biopsy proven acute rejection was also associated with a lower mental HRQoL. Although this association was not found among older KTR before [28], it has been previously described among younger KTR [28, 38]. We hypothesize that a rejection may reduce confidence in the transplant function and expectations for the future. Together with the accompanying hospital admission—a factor which was also associated with lower HRQoL in our study—there might be an increased perception of illness, leading to a diminished feeling of control over their disease. This could make KTR feel more like patients and heighten their awareness of their illness, factors that has been previously associated with lower mental HRQoL [39–41].

Therefore, it is very important that clinicians are aware of the relationship between the above mentioned determinants and low mental HRQoL, so any physical and mental health issues can be openly discussed and both medical and psychosocial support can be provided if necessary. This needs to be emphasized, because in particular mental health problems, like anxiety and depression, often go unrecognized and untreated in patients on renal replacement therapy [42, 43].

A history of dialysis was also associated with a lower physical HRQoL among older KTR. Although this is in contrast with findings in other studies amongst KTR [44, 45], a negative association between time on dialysis and post-transplant HRQoL has been described before [32]. This negative association might be—besides better patient and graft survival [46]—an additional reason to aim for pre-emptive kidney transplantation. The association of PTDM with reduced physical HRQoL has not been described in KTR before, but a negative impact of diabetes mellitus on HRQoL has been well documented in the general population [47]. It underscores the importance of interventions aimed at preventing and treating PTDM [48].

The finding that participants with a HRQoL score below the median before transplantation showed a greater increase in HRQoL after transplantation, while participants with a HRQoL score above the median HRQoL showed no difference in HRQoL after transplantation, might indicate that older KTR with a poor HRQoL benefit the most from a kidney transplantation regarding HRQoL. It is unlikely that this result is simply a reflection of regression to the mean, given the huge difference between pre- and posttransplant scores. Also, HRQoL of older KTR with scores above the median before transplantation does not decline; there is no significant change after transplantation.

These results can contribute to the discussion of the pros and cons of kidney transplantation in the older, shared-decision making and expectation management.

Strengths of this study are the availability of a broad variety of characteristics of both older patients waitlisted for kidney transplantation and older KTR, which allowed us to compare both groups extensively and to provide an overview of factors associated with HRQoL at 1 year after transplantation of older KTR. In addition, we were able to compare paired HRQoL scores of before and after transplantation in a subgroup of older KTR. Furthermore, given the high study participation rate of 81%, our study sample is likely representative for the older KTR population. Despite the lack of HRQoL data 1 year post-transplantation for 40 older, living, KTR with functioning grafts—who were consequently excluded from analyses—we believe these missing data had minimal impact on our overall findings. Although we found some minor differences between both groups, the differences that were found (such as a different prevalence of diabetes at baseline), were not independently associated with lower HRQoL 1 year after transplantation. Moreover, HRQoL prior to transplantation was comparable between both groups. Therefore, the absence of these data probably does not influence our results importantly.

However, we cannot exclude the possibility of selection bias. We only included older KTR with HRQoL data at 1 year after transplantation, excluding patients who died (*N* = 4) or suffered from graft failure (*N* = 2) within the first year after transplantation, although these numbers were quite small. A few other factors that may limit the external validity of our study need to be mentioned. The first one is the observational, single-center study design. Groups of waitlisted and transplanted patients were not completely equal or comparable as a result of this design, and our findings for the comparison of HRQoL among these groups should therefore be interpreted with caution. Secondly, a relatively limited number of patients—especially those with repeated HRQoL measurements—were included. Nevertheless, even with this sample size, we identified significant differences in HRQoL and statistically significant associated factors. Third, the median Charlson comorbidity index of our study population was relatively low. Although we did not observe an association between this index and HRQoL, it does mean that our findings may have limited generalizability to populations with more severe comorbidity. Fourth, our sample consisted of a relatively high percentage of KTR that was pre-emptively transplanted or had a living donor.

Furthermore, due to logistical reasons, we did not perform repeated measurements of HRQoL of the waitlisted patients, even though such assessments might have provided additional information. Ideally, results should be established in larger populations and re-evaluated at later time points.

In conclusion, our study shows the advantage of kidney transplantation among older KTR, with a significantly higher HRQoL 1 year after transplantation compared to before transplantation. KTR with the lowest HRQoL scores before transplantation showed the largest improvement of these scores after transplantation. The number of patient-reported immunosuppressive drug-related side effects was strongly associated with lower HRQoL of older KTR.

## Data Availability

The datasets presented in this article are not readily available because Public sharing of individual participant data was not included in the informed consent of the TransplantLines Biobank and cohort study, but data can be made available to interested researchers upon reasonable request by mailing to the data manager of the TransplantLines Biobank and Cohort study. Requests to access the datasets should be directed to datarequest.transplantlines@umcg.nl.

## Group members of Transplantlines Investigators

Coby Annema, Stephan JL Bakker, Stefan P Berger, Hans Blokzijl, Frank AJA Bodewes, Marieke T de Boer, Kevin Damman, Martin H de Borst, Arjan Diepstra, Gerard Dijkstra, Rianne M Douwes, Caecilia SE Doorenbos, Michele F Eisenga, Michiel E Erasmus, C Tji Gan, Antonio W Gomes Neto, Eelko Hak, Bouke G Hepkema, Marius C van den Heuvel, Frank Klont, Tim J Knobbe, Daan Kremer, Coretta van Leer-Buter, Henri GD Leuvenink, Marco van Londen, Willem S Lexmond, Vincent Ede Meijer, Hubert GM Niesters, Gertrude J Nieuwenhuis-Moeke, L Joost van Pelt, Robert A Pol, Robert J Porte, Adelta V Ranchor, Jan Stephan F Sanders, Marion J Siebelink, Riemer JHJA Slart, J Cas Swarte, Daan J Touw, Charlotte A te Velde-Keyzer, Erik AM Verschuuren, Michel J Vos, Rinse K Weersma.
