# Supplementary material for: Kidney Transplantation Improves Health-Related Quality of Life in Older Recipients
Source: Transpl Int. 2024 Apr 15;37:12071. doi: 10.3389/ti.2024.12071 (PMC11057459; doi:10.3389/ti.2024.12071)
Supplement: Supplementary file 1 [file DataSheet1.pdf]

SUPPLEMENTARY TABLES

Supplementary table 1 | Characteristics of older KTR with and without HRQoL data one year after transplantation.

Patient demographics	KTR without HRQoL data(N=40)	KTR with HRQoL data (N = 115)	P for difference
Male sex, *n* (%)	22 (55)	83 (73)	**0.045**
Age at time of Tx	70 ± 5	70 ± 4	0.416
White patients, *n* (%)	38 (95)	110 (96)	0.864
BMI, kg/m^2	28.3 ± 4.4	27 ± 4	0.084
Partner at time of Tx, *n* (%)	34 (85)	95 (83)	0.727
Children, *n* (%)	36 (90)	104 (90)	0.936
**Medical history**			
Dialysis before HRQoL assessment/Tx	29 (73)	69 (60)	0.158
Diabetes mellitus at time HRQoL assessment/Tx	12 (30)	17 (15)	**0.034**
Charlson Comorbidity Index score	3 [2 - 3]	3 [2-3]	0.140
Primary kidney disease, *n* (%)			0.386
Glomerulonephritis	5 (13)	19 (17)	
Interstitial nephritis	4 (10)	8 (7)	
Cystic kidney disease	5 (13)	17 (15)	
Other congenital/ hereditary disease	1 (3)	1 (1)	
Renal vascular disease, excluding vasculitis	5 (13)	26 (23)	
Diabetic nephropathy	5 (13)	7 (6)	
Other multisystem diseases	4 (10)	8 (7)	
Other	1 (3)	7 (6)	
Unknown	8 (20)	22 (19)	
**Transplant-specific characteristics**			
First kidney Tx, *n* (%)	38 (95)	106 (93)	0.165
Donor type, *n* (%)			0.415
Donation after cardiac death	18 (45)	41 (36)	
Donation after brain death	8 (20)	20 (17)	
Living donor	14 (35)	54 (47)	
Eurotransplant Senior Program donor, *n* (%)	18 (45)	42 (37)	0.343
ABO-incompatible transplantation, *n* (%)	1 (3)	7 (6)	0.681
Donor sex, *n* males (%)	24 (60)	64 (56)	0.633
Donor age, years	66 [53-70]	66 [62-70]	0.661
Induction therapy, *n* (%)			0.522
Basiliximab	38 (95)	108 (94)	
Antithymocyte globulin	1 (3)	2 (2)	
Alemtuzumab	0	2 (2)	
Rituximab	1 (3)	7 (6)	
None	0	3 (3)	
Immunosuppressive starting regimen, *n* (%)			0.569
TAC/MMF/prednisolone	37 (93)	108 (94)	
Cyclosporine/MMF/prednisolone	0	2 (2)	
TAC/EVR/ prednisolone	3 (8)	4 (4)	
Iscalimab (CFZ-533)/MMF/ prednisolone	0	1 (1)	
**HRQoL before transplantation**			
Mental HRQoL (MCS)	45.1 ± 9.4*	49.1 ± 8.4#	0.105
Physical HRQoL (PCS)	44.2 ± 8.1*	48.1 ± 8.0#	0.082

Normal distributed variables are presented as mean ± SD, not-normally distributed variables as median [IQR] and categorical data as number (valid %).

Abbreviations: EVR, everolimus; HRQoL, Health related quality of life; KTR, kidney transplant recipients; (K)Tx, (kidney)transplantation; MMF, mycophenolic acid; TAC, tacrolimus; MCS, mental component score of the SF-36 health-related quality of life questionnaire; PCS, physical component score of the SF-36 health-related quality of life questionnaire.

** Data available for 18 KTR. # Data available for 46 KTR.*

Bold values represent statistically significant associations (P <0.05).

Supplementary table 2 | Clinical outcomes one year after transplantation for older KTR with and without HRQoL data one year after transplantation.

Outcome	KTR without HRQoL data(N=40)	KTR with HRQoL data (N = 115)	P for difference
eGFR, ml/min/1.73m^2	45 ± 19	48 ± 16	0.311
eGFR < 30 ml/min/1.73m^2, n (%)	9 (23)	15 (13)	0.154
Hemoglobin, g/dL (SD)	12.4 ± 1.6	13.2 ± 1.6	**0.004**
Delayed graft function, n (%)	9 (23)	26 (23)	0.989
Rejection, n (%)	6 (15)	9 (8)	0.186
Post-transplant diabetes mellitus, n (%)	7 (18)	20 (17)	0.988
Any kind of malignancy, n of KTR with (%)	3 (8)	7 (6)	0.719
Any cardiovascular event, n of KTR with	0	3 (3)	0.569
Number of hospitalizations per KTR			0.107
No hospitalization	22 (55)	59 (51)	
1 hospitalization	8 (20)	30 (26)	
2 hospitalizations	3 (8)	19 (17)	
≥ 3 hospitalizations	7 (18)	7 (6)	
Number of infections in the first year after Tx per older KTR[#], *n* (%)			0.692
No infection	18 (45)	47 (41)	
1 infection	7 (18)	30 (26)	
2 infections	6 (15)	18 (16)	
≥ 3 infections	9 (23)	20 (17)	
CMV-primo infection in the first year after Tx, *n* (%)	4 (10)	7 (6)	0.476
BK viraemia in the first year after Tx, *n* (%)	11 (28)	25 (22)	0.457

Normal distributed variables are presented as mean ± SD, not-normally distributed variables as median [IQR] and categorical data as number (valid %).

Abbreviations: BCC, Basal cell carcinoma; eGFR, estimated glomerular filtration rate; KTR, kidney transplant recipient; SCC, Squamous cell carcinoma.

**One patient had a SCC and other cancer*

# Excluding CMV and BK viraemia

Bold values represent statistically significant associations (P <0.05).

Supplementary Table 3| HRQoL subdomain scores and summary scores of older patients waitlisted for kidney transplantation and of the age-matched general population.

SF-36 scores	Waitlisted older patients (N=145)	Age-matched general population (N=118**)**	P-value for difference waitlisted vs age-matched	Older KTR one year after Tx (N=115)	P-value for difference older KTR vs age-matched
**Subdomain scores of HRQoL**					
Mental health	78.9 ± 13.5	75.9 ± 17.3	**0.009**	81.3 ± 13.5	**<0.001**
Role limitations due to emotional problems	75.2 ± 37.0	82.9 ± 33.8	**0.007**	85.2 ± 30.0	0.41
Social functioning	72.5 ± 22.9	83.2 ± 23.7	**< 0.001**	84.0 ± 17.2	0.61
Vitality	57.4 ± 18.4	64.2 ± 22.0	**< 0.001**	67.7 ± 17.8	**0.035**
General health perceptions	47.3 ± 17.6	60.1 ± 23.9	**< 0.001**	62.4 ± 18.0	0.167
Bodily pain	79.3 ± 23.3	74.8 ± 28.0	**0.022**	86.4 ± 17.9	**<0.001**
Role limitations due to physical health	51.0 ± 44.1	69.1 ± 42.5	**< 0.001**	70.0 ± 38.2	0.80
Physical functioning	64.7 ± 24.2	66.7 ± 26.0	0.33	74.0 ± 20.6	**<0.001**
**Summary scores of HRQoL**					
Standardized mental component score	48.5 ± 8.4	50.0 ± 10.0	**0.037**	51.2 ± 7.7	0.10
Standardized physical component score	47.4 ± 8.5	50.0 ± 10.0	**< 0.001**	52.1 ± 7.2	**0.003**

Abbreviations: HRQoL, health-related quality of life; KTR, kidney transplant recipient; Tx, transplantation.
Bold values represent statistically significant associations (P <0.05).

Supplementary Table 4| HRQoL subdomain scores and summary scores of older patients waitlisted for kidney transplantation and of older KTR one year after transplantation.

SF-36 scores	Waitlisted older patients (N=145)	Older KTR one year after Tx (N=115)	Difference + pooled SD of difference	P-value for difference	Cohen's D
**Subdomain scores of HRQoL**					
Mental health	78.9 ± 13.5	81.3 ± 13.5	2.4 ± 13.5	0.16	0.18
Role limitations due to emotional problems	75.2 ± 37.0	85.2 ± 30.0	10.0 ± 34.1	**0.016**	0.29
Social functioning	72.5 ± 22.9	84.0 ± 17.2	11.5 ± 20.6	**<0.001**	0.56
Vitality	57.4 ± 18.4	67.7 ± 17.8	10.3 ± 18.1	**<0.001**	0.57
General health perceptions	47.3 ± 17.6	62.4 ± 18.0	15.1 ± 17.8	**<0.001**	0.84
Bodily pain	79.3 ± 23.3	86.4 ± 17.9	7.1 ± 21.1	**0.006**	0.34
Role limitations due to physical health	51.0 ± 44.1	70.0 ± 38.2	19.0 ± 41.6	**<0.001**	0.46
Physical functioning	64.7 ± 24.2	74.0 ± 20.6	9.3 ± 22.7	**0.009**	0.41
**Summary scores of HRQoL**					
Standardized mental component score	48.5 ± 8.4	51.2 ± 7.7	2.6 ± 8.1	**0.009**	0.32
Standardized physical component score	47.4 ± 8.5	52.1 ± 7.2	4.7 ± 8.0	**<0.001**	0.59

Abbreviations Tx, transplantation; KTR; kidney transplant recipients, SF-36, short-form 36.
Bold values represent statistically significant associations (P <0.05).

Supplementary Table 5| HRQoL subdomain scores and summary scores before and one year after transplantation for a subgroup of 46 older KTR with paired HRQoL data.

SF-36 scores	Older KTR before Tx (N= 46)	Older KTR one year after Tx (N=46)	Difference + SD difference	P-value for difference	Cohen's D
**Subdomain scores of HRQoL**					
Mental health	80.6 ± 13.5	83.5 ± 12.3	2.9 ± 13.8	0.16	0.21
Role limitations due to emotional problems	79.7 ± 31.8	85.5 ± 30.3	5.8 ± 40.6	0.38	0.14
Social functioning	72.6 ± 21.3	85.1 ± 17.4	12.5 ± 24.2	**0.001**	0.52
Vitality	57.5 ± 20.2	67.5 ± 19.6	10.0 ± 18.8	**0.001**	0.53
General health perceptions	48.2 ± 19.9	60.3 ± 18.7	12.2 ± 21.6	**<0.001**	0.56
Bodily pain	79.9 ± 24.7	87.2 ± 18.3	7.3 ± 28.2	0.09	0.26
Role limitations due to physical health	53.8 ± 42.5	72.8 ± 37.6	19.0 ± 51.4	**0.016**	0.37
Physical functioning	69.8 ± 23.0	77.1 ± 20.2	7.3 ± 17.3	**0.007**	0.42
**Summary scores of HRQoL**					
Standardized mental component score	49.1 ± 8.4	51.6 ± 7.5	2.5 ± 8.6	0.05	0.29
Standardized physical component score	48.1 ± 8.0	52.4 ± 6.7	4.3 ± 8.2	**0.001**	0.52

Abbreviations: Tx, transplantation; KTR; kidney transplant recipients; SF-36, short-form 36.
Bold values represent statistically significant associations (P <0.05).

Supplementary Table 6| HRQoL scores of older KTR before and after transplantation.

	Older KTR		P-value for difference	Older KTR		P-value for difference
	standardized MCS ≤ median before Tx (N=23)	standardized MCS ≥ median before Tx (N=23)		standardized PCS ≤ median before Tx (N=23)	standardized PCS ≥ median before Tx (N=23)	
Pre-transplant score	42.7 ± 6.9	55.4 ± 3.3	**<0.001**	41.6 ± 5.5	54.6 ± 3.4	**<0.001**
Post-transplant score	50.3 ± 7.2	52.8 ± 7.7	0.136	51.0 ± 7.2	53.7 ± 6.0	0.093
P-value for difference between pre-and posttransplant score	**< 0.001**	0.08	n/a	< 0.001	0.45	n/a
Difference in pre- and posttransplant score	7.6 ± 7.0	2.6 ± 6.7	**<0.001**	9.5 ± 6.8	2.6 ± 6.7	**<0.001**
Cohen's D for difference between pre- and posttransplant score	1.1	-0.4	n/a	1.4	-0.2	n/a

Abbreviations: KTR; kidney transplant recipients, Tx, transplantation; MCS, mental component score of the SF-36 health-related quality of life questionnaire; PCS, physical component score of the SF-36 health-related quality of life questionnaire.

Bold values represent statistically significant associations (P <0.05).

Supplementary Table 7 | Associations with mental and physical HRQoL at one year after transplantation among older KTR.

	Linear regression analyses with standardized MCS as dependent variable			Linear regression analyses with standardized PCS as dependent variable		
	Crude			Crude		
**Variables**	**St. β**	**95% CI**	**P**	**St. β**	**95% CI**	**P**
**Patient demographics**						
Female sex	-0.18	-0.36 to 0.00	0.05	-0.01	-0.20 to 0.17	0.90
Age	0.07	-0.12 to 0.26	0.46	-0.04	-0.23 to 0.15	0.67
BMI	0.12	-0.07 to 0.30	0.21	0.02	-0.17 to 0.20	0.87
Partner	0.16	-0.03 to 0.35	0.09	-0.00	-0.20 to 0.19	0.96
Children	-0.03	-0.21 to 0.16	0.79	0.00	-0.19 to 0.19	1.00
Education level						
Low	-0.25	-0.48 to -0.02	**0.037**	0.03	-0.21 to 0.28	0.78
Intermediate		reference			reference	
High	-0.10	-0.33 to 0.13	0.40	0.03	-0.21 to 0.27	0.80
Financial situation						
(Some) shortage of money	-0.02	-0.24 to 0.20	0.86	-0.04	-0.26 to 0.18	0.71
Just right		reference			reference	
(Some) money left	-0.09	-0.31 to 0.12	0.40	-0.24	**-0.47 to -0.04**	**0.022**
**Medical history**						
Pre-emptively transplanted .	0.01	-0.18 to 0.19	0.95	0.24	**0.07 to 0.43**	**0.008**
Diabetes mellitus pre-KTx	0.05	-0.14 to 0.24	0.60	0.04	-0.14 to 0.23	0.66
CCI pre-KTx	-0.13	-0.31 to 0.06	0.18	-0.11	-0.30 to 0.07	0.24
CCI 3 or higher pre-Tx	-0.18	-0.37 to -0.00	0.050	-0.17	**-0.35 to 0.02**	**0.08**
**Kidney transplant characteristics**						
Living donor	0.01	-0.18 to 0.20	0.91	0.04	-0.15 to 0.22	0.71
**Clinical course of the first year after Tx**						
eGFR	0.15	-0.03 to 0.34	0.10	-0.06	-0.24 to 0.13	0.54
eGFR <30 ml/min/1.73m^2	-0.16	-0.34 to 0.02	0.09	-0.09	-0.27 to 0.10	0.36
Hemoglobin		-0.02 to 0.35	0.08	0.19	**0.01 to 0.37**	**0.043**
Delayed graft function	-0.15	-0.18 to 0.03	0.11	-0.11	-0.30 to 0.07	0.22
Rejection in the first year after Tx	-0.31	**-0.48 to -0.13**	**<0.001**	-0.00	-0.19 to 0.19	0.97
Malnourished at one year after Tx	0.11	-0.08 o 0.31	0.25	-0.18	-0.38 to 0.02	0.08
Development of PTDM during the first year after Tx	-0.10	-0.29 to 0.08	0.28	-0.23	**-0.41 to -0.04**	**0.015**
Number of hospitalizations in the first year after Tx per older KTR	-0.16	-0.34 to 0.02	0.08	-0.15	-0.33 to 0.03	0.11
Any hospitalization in the first year after Tx	-0.19	**-0.37 to -0.00**	**0.045**	-0.18	-0.36 to 0.01	0.06
Number of infections in the first year after				-0.15	-0.33 to 0.04	0.11
Tx per older KTR	-0.34	-0.32 to 0.05	0.15			
Any infection in the first year after Tx	-0.08	-0.27 to 0.11	0.39	-0.12	-0.30 to 0.07	0.20
Number of side effects in the first year after Tx per older KTR	-0.4	**-0.55 to -0.20**	**<0.001**	-0.50	**-0.67 to -0.33**	**<0.001**

Data regarding educational level was missing in 7 older KTR; 19 older KTR had missing data regarding financial situation or did not want to tell their financial situation.

Abbreviations: MCS, mental component score of the SF-36 health-related quality of life questionnaire; PCS, physical component score of the SF-36 health-related quality of life questionnaire; CCI, Charlson Comorbidity Index; eGFR, estimated glomerular filtration rate; PTDM, post-transplant diabetes mellitus; Tx, transplantation.

Bold values represent statistically significant associations (P <0.05).
